# The Metabolic Syndrome and Its Components in African-American Women: Emerging Trends and Implications

**DOI:** 10.3389/fendo.2017.00383

**Published:** 2018-01-22

**Authors:** Trudy R. Gaillard

**Affiliations:** ^1^College of Nursing, University of Cincinnati, Cincinnati, OH, United States

**Keywords:** metabolic syndrome, African-American women, obesity, hypertension, type 2 diabetes

## Abstract

The Metabolic Syndrome (MetS) is recognized as a predictor of cardiovascular outcomes and type 2 diabetes (T2DM). The MetS is a constellation of clinical and metabolic risk factors that include abdominal obesity, dyslipidemia, glucose intolerance, and hypertension. There are ethnic and racial differences in the prevalence of MetS and its components. In general, African-Americans have lower prevalence of MetS when compared to whites, but suffer disproportionately from higher cardiovascular mortality and T2DM. Specifically, African-American women (AAW) have higher rates of T2DM and cardiovascular mortality despite a more favorable lipid and lipoprotein profile. This is paradoxical. However, there is a general upward trend in the prevalence of MetS in the US. The reasons are debatable, but could be multifactorial, including genetics and environmental factors. Thus, there is a need to understand the increasing trend in the MetS, its components, and the associated outcomes for AAW. Therefore, the purpose of this mini review is to (1) understand the increasing prevalence of MetS and its components in AAW and (2) provide suggestions for future prevention of cardiovascular disease and T2DM in AAW.

## Introduction

The metabolic syndrome (MetS) is a constellation of interrelated clinical and metabolic risk factors; including abdominal obesity, dyslipidemia, glucose intolerance, and hypertension that are associated with increased risk for cardiovascular disease (CVD) and type 2 diabetes (T2DM) (1–5). The mechanisms of MetS are not fully understood; however, it appears that insulin resistance is the major underpinning (6, 7). In fact, insulin resistance is related to obesity, T2DM, and hypertension all of which have dramatically increased over the last four decades. Ford et al. estimated the age-adjusted prevalence of the MetS to be 23.7% in the Third National Health and Nutrition Examination Survey (NHANES III) data (1988–1994), with similar prevalence for men (24%) and women (23.4%) (2). In the NHANES III, there were ethnic/racial differences in the prevalence of MetS, with African-American (AA) men having the lowest prevalence (16.4%) compared to white men (24.8%) and similar among AA women (AAW) (25.7%) and white women (22.8%) (2). In a recent report, Mozumdar and Liguori comparing the prevalence of the MetS in the NHANES III and NHANES 1999–2006 data found an increase in the age-adjusted prevalence of MetS from 29.2 ± 1.0 to 34.2 ± 0.7% (8). For AAW, the prevalence of MetS increased from 30.6 to 36.5%, respectively (8). This represented an absolute change of 5.9% and a relative change of 19.3% among AAW. The major increase in MetS for women was attributed to obesity [waist circumference (WC)] increased from 46.0 ± 1.4 to 58.0 ± 1.1%; hypertension 27.8 ± 0.9 to 36.6 ± 0.8%; hypertriglyceridemia 24.7 ± 1.2 to 27.6 ± 0.8%; hyperglycemia 24.2 ± 1.2 to 29.2 ± 1.0% (8). The authors found the greatest increase in MetS among young AAW, 20–39 years, which was attributed to increases in WC and hypertension (8). Moore et al. examined the increasing trends in MetS in the NHANES for three time-periods, 1988–1994, 1999–2006, and 2007–2012 and reported that MetS increased from 25.29, 24.99, and 34.17%, respectively (9). In this study, the prevalence of MetS for AAW increased from 14.48, 14.02 to 20.89%, compared to 15.28, 17.37 to 25.08% for white women, for the corresponding time-periods. There are several reasons for the increases in MetS among women; these include aging, increases in obesity, T2DM, hypertension, and physical inactivity. In addition, there were racial/ethnic differences in the components of MetS observed in the NHANES data. Because of the dipartites in T2DM and cardiovascular outcomes in AAW when compared to white women, it is of utmost importance to understand the trends of MetS and its components in AAW. Thus, the purpose of this Mini Review is to (1) understand the increasing prevalence of MetS and its components in AAW and (2) provide suggestions for future prevention of CVD and T2DM in AAW.

## Definition of the MetS

In the US, the National Cholesterol Education Program (NCEP), Adult Treatment Panel ATP III (NCEP-ATP) is used to define MetS (Table 1) (1). The American Diabetes Association and the American Association of Clinical Endocrinologist has modified this definition to include a lower reference for glucose intolerance (10, 11).

**Table 1:** Metabolic syndrome criteria in women according to the National Cholesterol Education Program (NCEP), Adult Treatment Panel ATP III (NCEP-ATP).

Parameter	Criteria
Elevated waist circumference	≥88 cm or 35 inches
Elevated triglycerides	≥150 mg/dL
Reduced HDL-C	<50 mg/dL
Elevated blood pressure (BP)	≥130 mmHg systolic BP
	≥85 mmHg diastolic BP
High fasting glucose	≥100 mg/dL

*Adapted from Ref. (1)*.

According to NCEP-ATP, three or more of the following criteria constitutes MetS in women; abdominal obesity WC ≥88 cm; Hypertriglyceridemia ≥150 mg/dL; Low HDL-C <50 mg/dL; elevated blood pressure (BP) ≥130/85 mmHg; elevated fasting glucose ≥ 100 mg/dL (Table 1). Recent studies have shown ethnic/racial differences in not only the MetS but also its components (2, 4, 8, 9).

## Components of the MetS in AAW

### Obesity

The prevalence of obesity has increased steadily over the past decades with over two-thirds of US adults being either overweight or obese (12–14). Obesity is clinically defined as body mass index (BMI) >30 kg/m^2^. However, data suggest that a more accurate reflection of true metabolic risk is central adiposity, assessed clinically by WC (15). Conversely, WC plays a critical role in the development of MetS and appears to antecede the development of other MetS components (4, 12, 14). Thus, the debate as to whether WC is superior to other anthropometric indices is ongoing and varies among gender and racial/ethnic groups (16).

The NCEP-ATP defines abdominal obesity as WC >88 cm in women and varies among racial/ethnic populations (1). Ford et al., using the NHANES III data, reported 46.3% of women had abdominal obesity (37.2% white and 44.6% black) (2). There has been an increasing trend in WC for AAW, according to NHANES, 1988–1994 (38.25%); 1999–2006 (56.7%); and 2007–2012 (68.8%) (9). Therefore two-thirds of AAW manifest abdominal obesity. In a study by our group, 35.5% of AAW had MetS (17). Most importantly, WC was the most common parameter to likely meet the MetS in our studies (17, 18). In the Jackson Heart Study (JHS), 76.5% of AAW had WC ≥88 cm (5). In another study of AAs and whites, 30–64 years, WC was the most powerful tool to predict MetS (19). Furthermore, Shen and colleagues reported that WC had the strongest association with health-risk indicators, followed by BMI and MetS components (15).

Another factor that contributes to MetS is intraabdominal visceral adiposity. There are ethnic/racial differences in visceral adipose tissue (VAT) and subcutaneous adipose tissue (SAT) (20–22). In general, VAT is more pathogenic and has a stronger association with insulin resistance and MetS (20–22). For the same BMI, AAW have lower VAT when compared to their white counterparts (20–23). However, AAW are more insulin resistant despite the lower VAT. This is paradoxical. In a study by Liu et al. examining SAT and VAT with cardiometabolic risk factors between AAW in the JHS and white women in the Framingham Heart Study (FHS) found that the associations between VAT were stronger in the FHS than in the JHS (20). These results may partly contribute to the apparent paradox of lower VAT in the setting of cardiometabolic risk factors in AAW when compared to their white counterparts. However, there were limitations in conduct of this study. First, there were different protocols for the CT-scan measurement of adipose tissue volume. Second, both studies were single center studies of AA and white women, thus the results cannot be generalizable. Third, these studies were cross-sectional and, therefore, the authors could not determine the causal relationship between abdominal VAT, SAT, and MetS. Of note, similar paradoxical relationships have been found in AA children (24), adolescence (25), young adults (26), and older adults in the Insulin Resistance Atherosclerosis Study (IRAS) (27) and Atherosclerosis Risk in the Community (ARIC) (28) studies. The reasons for the paradoxical relationship between VAT and obesity remain unknown, but may be related to other environmental and genetic factors. Thus, the consequences of VAT and cardiometabolic risk factors warrant further investigation in AAW.

### High Blood Pressure

The prevalence of hypertension among AAs is one of the highest is the world. Hypertension is more common in AAs than whites and occurs in approximately 41.2 vs 28%, respectively (29, 30). Data from NHANES demonstrate that the prevalence of hypertension in young adults, 20–40 years is twofold higher in AAs than in their white counterparts (9). The higher rates of hypertension in AAs contribute to the excess CVD morbidity and mortality in AAs with and without diabetes, lipid/lipoprotein disorders, or obesity (2–6, 8, 9, 18, 19, 24, 25, 30, 31). The American Heart Association reports that AAW suffer disproportionally from higher rates hypertension and CVD mortality when compared to white women (32). The exact causes of hypertension in AAW are unknown. However, it has been speculated that genetic and environmental factors, increased vascular reactivity, and increase renal sodium reabsorption are contributing factors (32, 33). Insulin resistance has been associated with BP in several populations; however, there is a very weak relationship in AAs when compared to whites (6, 7, 20, 21, 26–30). This is paradoxical in AAs.

There is evidence for increasing trends in BP in the NHANES cohort from 1988 to 1999 (33.92%), 1999 to 2006 (40.62%), and 2007 to 2012 (42.72%) (9). In addition, the prevalence of hypertension also increased among AAW for the same time-periods (38.5, 45.1, and 50.1%, respectively) (Table 2). Hence, Gaillard et al. found that 35.7% of AAW with family history of T2DM were hypertensive, and that increasing BP was associated with increasing prevalence of MetS and its components (31). Similarly, the JHS reported 73.1% of AAW were hypertensive (5). Given the havoc and the cardiovascular morbidity and mortality associated with hypertension, it is important to initiate early intervention strategies in AAW to control BP.

**Table 2:** Prevalence of metabolic syndrome (MetS) in US white and African-American female adults, National Health and Nutrition Examination Survey, 1988–2012.

Year	1988–1994		1999–2006		2007–2012	
Characteristic	White women	African-American (AA) women	White women	AA women	White women	AA women
MetS (%)	15.28	14.48	17.37	14.02	25.08	20.89
Elevated waist circumference (%)	35.9	48.6	54.4	65.2	58.8	68.3
Elevated triglycerides (%)	22.5	13.0	23.8	14.9	29.2	20.9
Reduced HDL cholesterol (%)	35.1	32.4	26.4	22.0	46.2	43.1
Elevated blood pressure (%)	31.1	38.5	39.3	45.1	48.8	50.1
Elevated fasting glucose (%)	22.7	24.5	15.9	19.2	22.2	24.5

*Adapted from Ref. (9)*.

### Glucose Intolerance and T2DM

Diabetes and prediabetes has become epidemic in the US affecting 29.1 million and 89 million adults, in the US, respectively (34). Patients with prediabetes and T2DM have greater cardiovascular risks and events (i.e., congestive heart failure, stroke) as well as the associated morbidity and mortality than in non-diabetic subjects (34, 35).

The Center for Disease Control and Prevention report that risk of diabetes is 77% higher among AAs than among whites.^1^ In fact, AAW are twice as likely to be diagnosed with T2DM when compared to whites (36, 37). The reasons for the higher prevalence of diabetes in AAs are associated with genetics and environmental factors (i.e., low physical activity, poor dietary/nutrition practices, stress). Most importantly, the fundament lesion for T2DM is beta-cell dysfunction and insulin resistance, which are higher in AAW when compared to white women (38–41). Similar findings have been demonstrated in AA children (29, 41), young adults (26), and middle aged adults in the IRAS (27) and ARIC (28) studies.

Type 2 diabetes is an outcome of MetS. Therefore, individuals with T2DM have higher prevalence of MetS that those without MetS. AAs have a disproportionate higher burden of T2DM, independent of BMI when compared to whites (20, 28, 36, 37, 39). The higher prevalence of glucose intolerance, prediabetes, and T2DM has been attributed in part to the greater insulin resistance in AAs compared to whites (37–42). In particular, AAW, with and without glucose intolerance, manifest greater insulin resistance than their white counterparts (36–39). In this regard, interventions such as the Diabetes Prevention Program (DPP) are effective strategies to improve insulin resistance and prevent progression to diabetes (43). In this regard, 53% of subjects in the DPP had MetS (44).

In the NHANES, the prevalence of fasting glucose meeting the NCEP-ATP criteria did not significantly change over the three time-periods, including AAW (Table 2). The lack of remarkable changes in fasting glucose over time is surprising in view of the increases in obesity among AAW. Thus, obesity is considered a major contributor to glucose dysregulation and insulin resistance. Nevertheless, it may be possible that AAW may be more sensitive to adverse changes in obesity given their higher prevalence of diabetes compared to whites (45). In addition, changes in the categories and classification of glucose intolerance, prediabetes, and T2DM may also contribute to the lack of significant increases in T2DM (10, 42, 46). In this regard, Bullard et al. examined the estimated prevalence of prediabetes in the NHANES (1999–2010) and reported a 21% increase (46). The authors reported the greatest increase was found among adolescent girls and women.

Prediabetes is a precursor to T2DM and is associated with higher cardiovascular risk and events in several populations (36–42, 46). In this regard, Osei et al. reported that first-degree relatives of AAs with normal glucose tolerance who progressed to impaired glucose tolerance and T2DM after 6 years manifest insulin resistance, beta cell dysfunction, and weight gain before diagnosis of T2DM compared to those who did not progress to T2DM (47). In the IRAS study, D’Agostino et al. examined whether CVD risk factors predicted future development of T2DM in 5 years (48). The authors concluded that for each CVD risk factor (hypertension, hypertriglyceridemia, low HDL-C, and impaired glucose tolerance) there was a doubling of risk for conversion to diabetes when compared to those without risk factors (48). Most importantly, impaired glucose tolerance was the strongest risk factor associated with conversion to diabetes in this multiethnic population, which included AA (39).

### Dyslipidemia

Dyslipidemia is defined as abnormalities in cholesterol, low density lipoprotein cholesterol (LDL-C), hypertriglyceridemia, and HDL-C (1, 3, 4, 6, 7, 20, 32, 49). These alterations in lipids/lipoproteins have been associated with increased cardiovascular morbidity and mortality (i.e., myocardial infarction, stroke, and heart failure). Thus, dyslipidemia constitute a leading cause of deaths in the US. With respect to the MetS, high triglycerides and low HDL-C are major components. The traditional lipid profile found in MetS has been attributed to insulin resistance and obesity. Major epidemiological studies have confirmed this atherogenic profile in several ethic/racial populations (49–51). However, AAs have lower triglycerides and higher HDL-C levels than whites (48–50). Thus, theoretically, this favorable lipid profile should protect AAs from coronary heart disease. Surprisingly, AAs with insulin resistance have lower triglycerides and higher HDL-C when compared to whites (15–18, 26–30). Thus, there is a paradoxical relationship between lipids/lipoproteins and CVD in AAs (6). This paradox has been found in AA children in the Bogalusa Heart Study (25, 41), in young adults in the CARDIA study (26), and for adults in the ARIC (28), IRAS (27, 37), and JHS (5).

The increasing prevalence of obesity in the US predisposes the population to alterations in lipids/lipoproteins. In the NHANES—1988–1994, 1999–2006, 2007–2012, there has been a modest increase in the prevalence of elevated triglycerides and low HDL-C levels, including AAW (Table 2). The reasons are uncertain, but could reflect the increasing rate of obesity, lack of physical activity, and higher caloric consumption among AAW (52). In addition, several studies have examined the subclasses of lipids/lipoproteins using nuclear magnetic resonance (NMR) technique to study the atherogenic properties of the lipoprotein concentration and particle size. Traditionally, measured LDL-C levels are positively and HDL-C levels are negatively associated with coronary heart disease in whites, but it remains controversial in blacks (53–57). In contrast, NMR-derived HDL particles inversely correlated with incident coronary heart disease in all populations, including Blacks (56, 57). Thus, we have previously demonstrated that AAW have more favorable NMR-derived lipoprotein profiles (53). But these findings cannot explain the paradoxically higher CVD mortality in AAW, thus requires further elucidation (6, 53–57).

### Genetics of the MetS

The MetS consists of five components that individually may be genetically determined. Thus, several authorities have argued and debated if there is a single genetic entity associated with MetS (58–62). Moreover, other authorities have argued that MetS is a sum of its components and a single genetically determined Mets does not exist (60–63). Also, there is no known genetic mutation that has been identified as a genetic predictor or marker for MetS. Furthermore, there has not been a single nucleotide polymorphism identified for MetS (58–63). Given the current scanty literature on this issue, there is urgent need to conduct more genetic studies in AAs and ethnic/racial populations with MetS.

### Prevention of Met Syndrome

The major determinant of the components of MetS is obesity, which is associated with insulin resistance. Thus, weight loss has been recommended for managing and preventing MetS. In this context, the DPP, the largest diabetes primary intervention program of multiethnic/multiracial US adults with prediabetes has been very effective in preventing progression to T2DM (43). In the DPP, 53% of patients had MetS (44). Most remarkable, the DPP recommended modest lifestyle changes, specifically 7% weight loss, combining caloric restriction (low fat) and increases in physical activity (150 min/week). These modest lifestyle recommendations resulted in reduction in the development of T2DM by 58% in all the populations, including AAs compared to 31% in those receiving metformin (43). The success of this program has been adapted by the Centers for Disease Control and Prevention and other health-care organizations (64, 65). Thus, the benefits of DPP extend beyond weight loss and included lower glucose, lipids/lipoproteins levels and improved BP all of which are components of MetS. It is worthy to note that the improvement in MetS components had been sustained for at least 20 years in individuals who participated in the DPP program (44).

### Strengths and Limitations

In this mini review, we examined the literature and data for trends in the MetS and its components from NHANES 1988 to 2012 (2–9). The strength in this data is the large sample size of US represented adults and the ability to compare sequential data from multiple studies. A major limitation is that we did not perform analysis of the raw data nor was this a meta-analysis. Therefore, the data consist of only literature review with emphasis on AAW. Therefore, any conclusion is tentative and would require validation studies. In addition, we did not measure other factors such as, inflammation, oxidation, socioeconomic status, and environmental factors that are also known to be associated with the MetS in other large epidemiological studies (66–69). Moreover, there is only limited information on genetics and MetS in AAW. Other studies examining the contributions of these factors in AAW are warranted.

In summary, this mini review, discussed the increasing prevalence of MetS and its components in the NHANES data from 1988 to 2012. The data demonstrate and increase in MetS among US and AAW. We conclude that the increasing prevalence of obesity and its associated risk factors contribute to the increases in MetS in the US and in AAW. Given these observations, we believe intervention strategies (i.e., DPP) aimed at reducing obesity should be a national priority in combating MetS and its associated cardiovascular outcomes and T2DM, especially among AAW.

## Author Contributions

The author has developed the ideas and content of the manuscript.

## Conflict of Interest Statement

The author declares that the research was conducted in the absence of any commercial or financial relationships that could be construed as a potential conflict of interest.

## Footnotes

^1^https://www.cdc.gov/chronicdisease/resources/publications/aag/pdf/2016/reach-aag.pdf.
